# Migrant female sex workers working at the Sino-Vietnamese border for a short time have a higher risk of HIV transmission: a consecutive cross-sectional study

**DOI:** 10.1186/s12981-020-0260-0

**Published:** 2020-02-07

**Authors:** Yu Zhang, Bingyu Liang, Deping Liu, Guangwu Wei, Shide Mo, Aidan Nong, Chuanyi Ning, Yanyan Liao, Junjun Jiang, PeiJiang Pan, Yuan Yang, Ning Zang, Dinh Vanphu, Nguyen Van, Li Ye, Hao Liang, Jiegang Huang

**Affiliations:** 1 Guangxi Key Laboratory of AIDS Prevention and Treatment & Guangxi Universities Key Laboratory of Prevention and Control of Highly Prevalent Disease, School of Public Health, Guangxi Medical University, Nanning, 530021 Guangxi China; 2 Guangxi Collaborative Innovation Center for Biomedicine, Life Science Institute, Guangxi Medical University, Nanning, 530021 Guangxi China; 3 Chongzuo Center for Disease Control and Prevention, Chongzuo, 532200 Guangxi China; 4 Fangchenggang Center for Disease Control and Prevention, Fangchenggang, 538000 Guangxi China; 5 HaTinh Medical College, Hà Tĩnh, HaTinh Province Vietnam

**Keywords:** AIDS, FSWs, Sino-Vietnamese border, Risk for HIV infection

## Abstract

**Objectives:**

For migrant female sex workers (FSWs) at the Sino-Vietnamese border, the impact of work time in their current location on the spread of HIV/AIDS is not clear.

**Methods:**

Data were collected from the Sino-Vietnamese border cities of Guangxi, China. Migrant FSWs working in these cities were studied. FSWs who worked less than 6 months in their current location were assigned to the short-term work group (ST FSWs), and FSWs who worked equal to or longer than 6 months in their current location were assigned to the long-term work group (LT FSWs). Logistic regression was performed to examine the impact of work time in the current location and factors associated with HIV infection.

**Results:**

Among the 1667 migrant FSWs, 586 (35.2%) and 1081 (64.9%) were assigned to the ST FSW and LT FSW groups, respectively. Compared to LT FSWs, ST FSWs were more likely to be of Vietnamese nationality, be less than 18 years old when they first engaged in commercial sex work, and have a low-level of HIV-related knowledge and had higher odds of using condoms inconsistently, having more male clients, having no regular male clients, and having a history of male clients who used aphrodisiacs but lower odds of receiving free condoms distribution and education/HIV counselling and testing programme. The analysis of factors associated with HIV infection revealed that Vietnamese FSWs, less than 18 years old when they first engaged in commercial sex work, having no regular male clients, and having lower average charge per sex transaction were correlated with HIV infection.

**Conclusion:**

FSWs with short-term work at the Sino-Vietnamese border had a higher risk of risky sex and were correlated with HIV risk factors. Vietnamese FSWs were at higher risk of HIV infection, and they were more likely to have short-term work. More targeted HIV prevention should be designed for new FSWs who recently began working in a locality to further control the spread of HIV, particularly cross-border FSWs.

## Introduction

Recently, heterosexual behaviour has become the predominant mode of HIV transmission in China, and it accounts for greater than 60% of all HIV infections [1, 2]. Female sex workers (FSWs) are at a high risk of HIV infection via heterosexual behaviour, acting as the “bridge” population of HIV transmission among the general population. The prevalence of HIV is high in FSWs in the country’s border region [3–5]. Guangxi is a Vietnam-bordered province in China, and it has the second-highest HIV infection rate in China [6]. The prevalence of HIV among FSWs in this province is over 1% [7, 8]. Simultaneously, a study conducted in other border provinces of Vietnam also reported a high prevalence of sexually transmitted infections among FSWs [9]. Due to the convenience of travel, cross-border trade and exchange activities between China and Vietnam are frequent in cities at the border of Guangxi. Numerous Vietnamese female immigrants cross the border into Guangxi and engaged in sex work each year and become the workforce of the thriving sex industry in the border regions of China [10].

FSWs often move their residence to earn more or avoid being recognized for privacy and illegal work purposes [11]. It was reported that FSWs spent a median of 6 months working in one location [12]. In China, approximately 62.3–95% of FSWs are immigrants [11], and approximately 43.3% move to another city within 1 year in some areas [4]. Thus, the mobility of FSWs in border regions might promote the spread of HIV/AIDS from regions along the border to non-bordered regions, making it more difficult to control. Migrant FSWs in new locations have often been reported to face several vulnerabilities, such as sexual violence, debt, and poor working or living conditions [13–15]. They often lack the ability to negotiate condom use with clients because of their low economic status [14]. Furthermore, because of language problems and ostracization by healthcare services, migrant FSWs in a new environment often lack communication and contact with local communities and are less likely to get access to health services [14]. Given the large number of migrant FSWs, it is essential to consider and adopt more targeted measures to further control the spread of HIV/AIDS among migrant FSWs. Previous studies generally assessed the impact of migration on HIV-related behaviour by considering all immigrant FSWs as a whole, despite their different lengths of work after they had arrived at their current location or only in light of their frequency of changing their residence in a past period [11, 13–17]. A study conducted in India found that FSWs who spent shorter average durations of stay in a visit were more likely to use condoms inconsistently [13]. Nevertheless, among migrant FSWs, the impact of work time after arriving at their current location on HIV transmission is seldom reported. Details about the impact of migrant FSWs’ work time in their current location on HIV-related factors and HIV status need to be further studied to provide evidence for better measures to prevent the spread of HIV/AIDS.

In this study, data of migrant FSWs were collected from China’s National Sentinel Surveillance (NSS) in two Sino-Vietnam border cities of Guangxi, China. Migrant FSWs in our study were divided into two groups depending on their length of sex work in their current location. We aimed to compare difference in the characteristics between these two groups and study the impact of the length of sex work in their current location and factors associated HIV infection.

## Methods

### Study setting

Data derived from the NSS in two Sino-Vietnam border cities of Guangxi, China (Fangchenggang City and Chongzuo City, a total of 11 jurisdictions, including 2 cities, 6 counties and 3 districts) were collected consecutively from 2016 to 2018. Chongzuo and Fangchenggang, two cities directly bordering Vietnam, cover areas of 17,331 and 6239 square kilometres, respectively. In 2016, Chongzuo had a permanent population of 2,069,100, and Fangchenggang had a permanent population of 929,000 [18]. With the establishment of the China-ASEAN free trade area (CAFTA) and the development of One Belt And One Road project, border trade and tourism between China and Vietnam have developed rapidly. The rapid economic growth has attracted women in Vietnam to immigrate to China to look for work opportunities. For women with low skills or who are eager for quick financial return, some of them are eventually involved in sex work [19].

### Study design and data collection

A cross-sectional survey of female sex workers was conducted by the local Centre for Disease Control and Prevention (CDC) from April to July each year. A venue-based sampling frame was generated according to a map of commercial sex establishments maintained by the local CDC. The venues of the two cities were sampled randomly based on the venue-based sampling frame generated. The venues included sauna/bath centres, nightclubs, karaoke halls, ballrooms, bars, hotels, hostels, hair salons, foot bath centres, restaurants and streets. Then, the FSWs in these venues were recruited via cluster sampling. The details of sampling and recruitment have been described elsewhere [20].

The study population needed to meet the following criteria: (1) female aged 16 years or older; (2) self-reported receipt of payment from a sex transaction in the past 6 months; (3) able to provide verbal and written consent; and (4) registered residence outside Guangxi. Before investigation, all participants were informed of the purpose and nature of the study, the steps of investigation, sensitivity of questions, confidentiality of the investigation, and payment for participation. Moreover, all participants signed an informed consent form. Participants were asked to attend face-to-face interviews and help complete a questionnaire conducted by local CDC staff. Chinese participants were interviewed in Mandarin. For Vietnamese participants, some were able to understand and were fluent in Mandarin, while other participants were not capable. The madam would translate for participants who were not familiar with Mandarin to help them complete the questionnaires. After completing the survey, participants received 50 RMB for compensation. The study was reviewed and approved by the Human Research Ethics Committee of Guangxi Medical University (ethical review No. 2013-130).

Up to 200 participants whose registered residence was outside Guangxi were recruited annually from Fangchenggang in 2017 to 2018. Approximately 400 participants whose registered residence was outside Guangxi were recruited annually from Chongzuo in 2016 to 2018.

## Measures

### Demographic background

The demographic information in the questionnaire included (1) current age, (2) marital status, (3) nationality (4) years of education, (5) total duration of sexual work, (6) length of sex work in current location, (7) types of working venues, (8) the average charge per sex transaction, and (9) the age when they first engaged in sex work. The total duration of sex work referred to the period from the beginning of commercial sex work to the time of the survey. The length of sex work in the current location referred to the period of sex work from their arrival at the current jurisdiction until the time of the survey, and the responses included less than 1 month, 1 to 6 months, 6 to 12 months, or longer than 12 months. Participants were assigned to the short-term work group or long-term work group depending on their length of sex work after their arrival in the current location. FSWs who worked less than 6 months in their current location were assigned to the short-term work group (ST FSWs), while FSWs who worked equal to or longer than 6 months in their current location were assigned to the long-term work group (LT FSWs). Working venues were divided into high-level venues, middle-level venues, and low-level venues. Participants who worked in bath rooms, night clubs or dancing halls and earned more than 200 RMB per sex transaction were considered to work in high-level venues; participants who worked in hotels, footbath salons or bars and earned 100 to 200 RMB per transaction were regarded as working in a middle-level venue; and participants who worked in hostels, hair salons or the street and earned less than 100 RMB per transaction were regarded as working in a low-level venue.

### HIV-related knowledge

Eight questions related to HIV in the questionnaire were used to estimate the level of HIV-related knowledge, including the transmission route, HIV treatment, and legal knowledge. Each question had a yes, no, or unknown response option. For each response, one point was awarded for a correct answer, and the points for each question were summed to yield a total score. Participants with a cumulative score of six or more points were considered to have a high-level of HIV-related knowledge, and participants with less than six points were considered to have a low-level of HIV-related knowledge.

### HIV-related behaviour and access to preventive intervention services

The following set of questions were used to evaluate HIV-related behaviour: (1) consistent condom use in the past month, which referred to the use of condoms each time they had sex; (2) lifetime illicit drug use, which referred to the previous use (intravenously or orally) of any illicit drugs; (3) the number of male clients in the past month; (4) the history of male clients who used an aphrodisiac during sex, which was defined as previously having male clients who occasionally or frequently used an aphrodisiac during sex; and (5) whether they had regular male clients in the past month, which was defined as whether they had known male clients who had paid for sex with them. Questions about access to preventive intervention services included (1) whether they received free condom distribution and education/HIV counselling and testing programme in the past year and (2) whether they participated in peer education in the past year.

### HIV testing

Qualified nurses in the local CDC tested blood specimens from all participants for the HIV antibody. According to the national AIDS sentinel surveillance programme in China, for surveillance purposes, enzyme-linked immunosorbent assays (ELISAs) were utilized for HIV preliminary screening and retesting, and HIV-1 western blot tests were performed for confirmation. The following test procedures were used. In HIV preliminary screening, human immunodeficiency virus (HIV1 + 2) antibody detection kit (Beijing Wantai Biological Pharmacy Enterprise Co., Beijing, China) was used. Participants with a negative result in HIV preliminary screening were considered HIV-seronegative and did not need to be retested. By contrast, samples from participants with a positive result in the HIV screening test were retested using an ELISA reagent from a different manufacturer (Multispot HIV-1/HIV-2 Rapid Test, Bio-Rad Laboratories Inc., Hercules, California, USA). Participants with a negative result on the retest were considered HIV-seronegative without a confirmation test. If a positive result was found on retesting, then HIV-1 western blot test (Diagnostics HIV Blot 2.2, MP Biomedicals Asia Pacific Pte Ltd., Singapore) was conducted for confirmation. Participants with a positive result in the confirmation test were regarded as HIV-seropositive, and participants with a negative result in the confirmation test were regarded as HIV-seronegative.

### Data analysis

All collected questionnaire data were input into Microsoft Excel (Microsoft Corporation, Redmond, USA), and participants with incomplete information were removed. SPSS Statistics 17.0 (SPSS Inc., Chicago, IL, USA) was used to carry out chi-squared tests, t-tests, and logistic regression analysis. Chi-squared tests and t-tests were performed to compare differences in demographic characteristics, HIV-related behaviour and access to preventive intervention services between ST and LT FSWs. The chi-squared test was used for categorical variables, and the t-test was used for continuous variables. Logistic regression was performed to analyse the impact of length of sex work in current location and factors associated with HIV infection. In the analysis of impact of length of sex work in current location, the length of sex work in current location was used as the independent variable, and HIV-related knowledge, HIV-related behaviour, and access to preventive intervention services were used as dependent variables to estimate the impact of length of sex work in current location adjusted by other covariates (current age, marital status, nationality, years of education, types of working venues, total duration of sex work, the level of HIV-related knowledge, age when they first engaged in commercial sex work, and average charge per sex transaction). In the analysis of factors associated with HIV infection, HIV infection was used as the dependent variable, and all other factors were used as independent variables for unadjusted and adjusted analysis.

## Results

A total of 1702 questionnaires were obtained. A total of 35 questionnaires had incomplete information, and 1667 questionnaires had complete information. Compared to results without removing incomplete questionnaires, the proportion of demographic characteristics, HIV-related behaviour, and access to preventive intervention services between ST FSWs and LT FSWs had slight changes after incomplete information questionnaires were deleted, but the results of difference comparison between ST FSWs and LT FSWs remained unchanged. Results from the analysis of the impact of length of sex work in the current location and analysis of factors associated with HIV infection also remained unchanged after removing incomplete questionnaires.

### Demographic characteristics between ST FSWs and LT FSWs

Among all the FSWs, 586 (35.2%) were assigned to the ST group, and 1081 (64.9%) participants were assigned to the LT group. There were 22 HIV-positive cases in all FSWs, and the numbers of HIV-positive cases in the ST group and the LT group were 4 (1.4%) and 18 (1.3%) respectively. The overall rate of HIV-positive cases was 1.3%. Compared with the LT FSWs group, the ST FSWs group was more likely to be younger (30 years vs. 39 years, P < 0.001), be unmarried or divorced/widowed (34.0% vs. 24.0%, P < 0.001), be of Vietnamese nationality (58.7% vs. 27.9%, P < 0.001), have over 9 years of education (21.3% vs. 4.4%, P < 0.001), work in medium-level (54.8% vs. 28.2, P < 0.001) and high-level venues (5.0% vs. 3.5%, P < 0.001), have a low-level of HIV-related knowledge (10.2% vs. 4.5%, P < 0.001), and be less than 18 years old when they first engaged in commercial sex work (5.5% vs. 0.9%, P < 0.001). Additionally, the total duration of sex work of the ST FSWs was shorter than that of the LT FSWs (16 months vs. 48 months, P < 0.001). There was no significant difference in average charge per sex transaction or HIV status between these two groups (Table 1).

**Table 1:** Demographic characteristics of ST FSWs and LT FSWs

Variables	Total (N = 1667, %)	ST FSWs (N = 586, %)	LT FSWs (N = 1081, %)	*Χ*^*2*^*/t*	P value
Current age (years)				12.7^a^	< 0.001
Median (IQR)	1667 (100%)	30 (24, 38)	39 (31, 45)		
Marital status				78.115	< 0.001
Unmarried/divorced/widowed	456 (27.4)	199 (34.0)	257 (24.0)		
Married/Cohabiting	1211 (72.6)	387 (66.0)	824 (76.0)		
Nationality				151.545	< 0.001
Chinese	1021 (61.3)	242 (41.30)	779 (72.1)		
Vietnamese	646 (38.7)	344 (58.70)	302 (27.9)		
Years of education				116.562	< 0.001
≤ 9	1494 (89.6)	461 (78.7)	1033 (95.6)		
> 9	173 (10.4)	125 (21.3)	48 (4.4)		
Types of working venues				124.326	< 0.001
Low-level	974 (58.4)	236 (40.2)	738 (68.3)		
Medium-level	626 (37.6)	321 (54.8)	305 (28.2)		
High-level	67 (4.0)	29 (5.0)	38 (3.5)		
Total duration of sex work (months)					
Median (IQR)	1667 (100%)	16 (4, 37)	48 (24, 72)	14.173^a^	< 0.001
The level of HIV-related knowledge				20.246	< 0.001
High-level	1558 (93.5)	526 (89.8)	1032 (95.5)		
Low-level	109 (6.5)	60 (10.2)	49 (4.5)		
Age when they first engaged in commercial sex work (years)				31.830	< 0.001
< 18	42 (2.5)	32 (5.5)	10 (0.9)		
≥ 18	1625 (97.5)	554 (94.5)	1071 (99.1)		
Average charge per sex transaction (RMB)				0.984	0.325
Median (IQR)	1667 (100%)	100 (80, 100)	100 (80, 120)		
HIV status				0.014	0.905
HIV-seronegative	1645 (98.7)	578 (98.6)	1067 (98.7)		
HIV-seropositive	22 (1.3)	8 (1.4)	14 (1.3)		

^a^Refers to corrected t test

### HIV-related behaviour and access to preventive intervention services between ST FSWs and LT FSWs

**Fig. 1:**
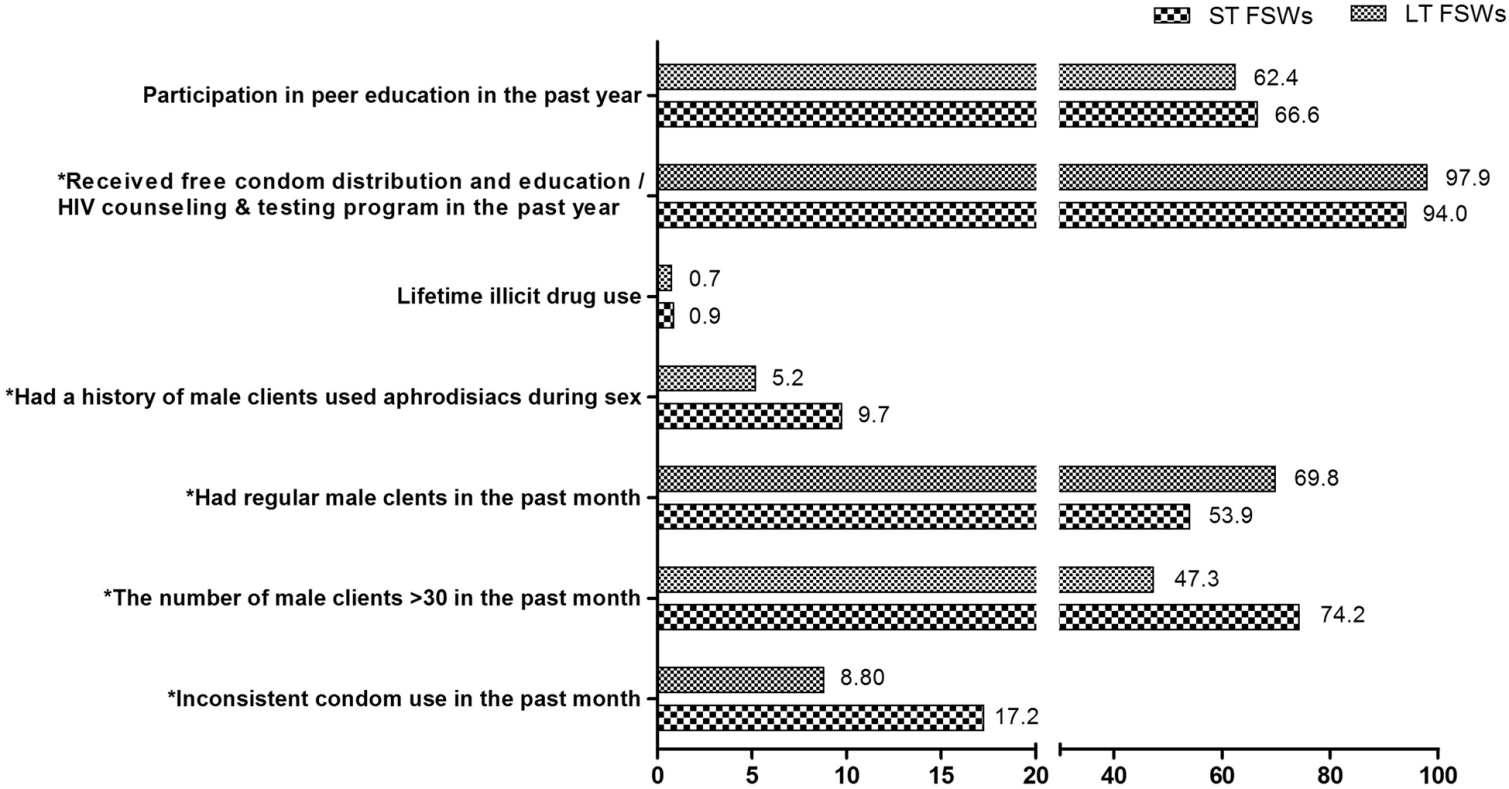
HIV-related behavior and access to preventive intervention services between the ST FSWs and LT FSWs. The proportion of inconsistent condom use in the past month, having more than 30 male clients in past month, having regular male clients in past month, having a history of male clients used aphrodisiacs, lifetime illicit drug use, not receiving free condom distribution or HIV counselling and testing programs in the past year, and no participation in peer education in the past year in both groups were calculated. Chi-Square tests were performed to compare the differences between the ST FSWs and the LT FSWs. *P < 0.05 between the two groups

Compared to LT FSWs, ST FSWs were more likely to use condoms inconsistently in the past month (17.2% vs. 8.8%, P < 0.001), have more than 30 male clients in the past month (74.2 vs. 47.3, P < 0.001), and have a history of male clients who used aphrodisiacs during sex (9.7 vs. 5.2, P < 0.001) but were less likely to have regular male clients in the past month (53.9% vs. 69.8%, P < 0.001) and have received free condoms or education/HIV counselling and testing programme in the past year (94.0% vs. 97.9%, P < 0.001). The proportion of lifetime illicit drug use and participation in peer education in the past year showed no significant difference between the two groups (Fig. 1, Additional file 1: Table S1).

### The impact of ST FSWs on HIV-related knowledge, HIV-related behaviour, and access to preventive intervention services

**Fig. 2:**
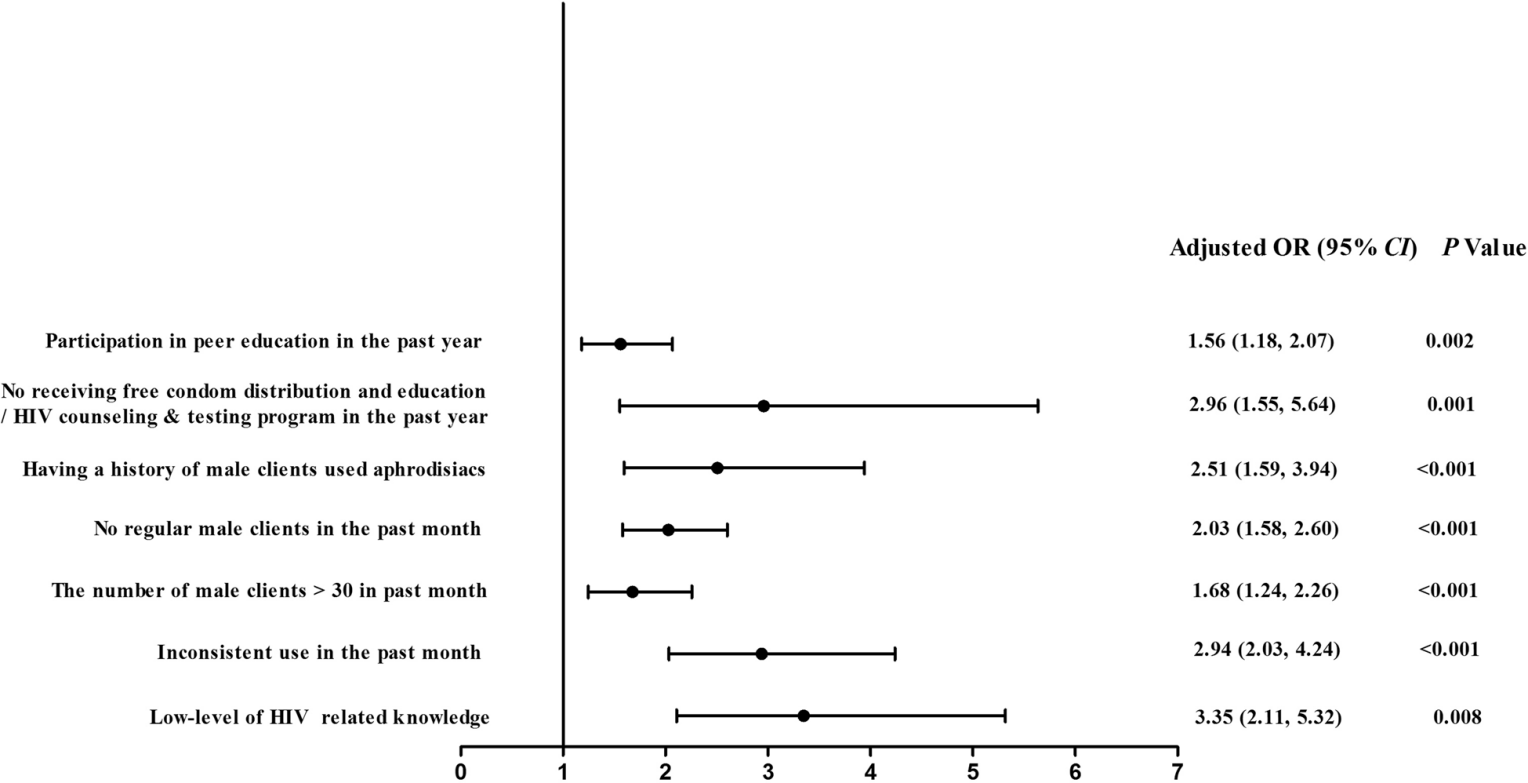
The impact of ST FSWs on HIV-related knowledge, HIV-related behaviour, and access to preventive intervention services. After adjustment, ST FSWs had significantly higher odds of having a low-level of HIV-related knowledge, inconsistent condom use in past month, having > 30 male clients in past month, having no regular male clients in past month, having a history of male clients used aphrodisiacs, not receiving free condom distribution and education/HIV counseling and testing program in the past year, and participation in peer education in the past year

As a result, compared with the LT FSWs, the ST FSWs had 3.35 times the odds of having a low-level of HIV-related knowledge (OR: 3.35, 95% CI 2.11–5.32, P = 0.008), 2.94 times the odds of using condoms inconsistently in the past month (OR: 2.94, 95% CI 2.03–4.24, P < 0.001), 1.68 times the odds of having > 30 male clients in the past month (OR: 1.68, 95% CI 1.24–2.26, P < 0.001), 2.03 times the odds of having no regular male clients in the past month (OR: 2.03, 95% CI 1.58–2.60, P < 0.001), 2.51 times the odds of having a history of male clients who used aphrodisiacs (OR: 2.51, 95% CI 1.59–3.94, P < 0.001), 2.96 times the odds of not receiving free condoms distribution and education/HIV counselling and testing programme in the past year (OR: 2.96, 95% CI 1.55–5.64, P = 0.001), and 1.56 times the odds of participating in peer education in the past year (AOR: 1.56, 95% CI 1.18–2.07, P < 0.001). No association was found between length of sex work in the current location and lifetime illicit drug use (Table 2, Fig. 2). Detailed results are shown in Additional file 1: Tables S2–S5).

**Table 2:** The impact of ST FSWs on HIV-related knowledge, HIV-related behaviour, access to preventive intervention services

Dependent variables	Adjusted OR^a^ (95% *CI*)	P value
HIV-related knowledge		
The level of HIV-related knowledge		
High-level	Referent	
Low-level	3.35 (2.11, 5.32)	0.008
HIV-related behaviour		
Consistent condom use in past month		
Yes	Referent	
No	2.94 (2.03, 4.24)	< 0.001
The number of male clients in the past month		
≤ 30	Referent	
> 30	1.68 (1.24, 2.26)	< 0.001
Had regular male clients in the past month		
Yes	Referent	
No	2.03 (1.58, 2.60)	< 0.001
Had a history of male clients used aphrodisiacs		
No	Referent	
Yes	2.51 (1.59, 3.94)	< 0.001
Lifetime illicit drug use		
Yes	Referent	
No	1.21 (0.32, 4.54)	0.778
Access to preventive intervention services		
Received free condom distribution and education/HIV counseling and testing program in the past year		
Yes	Referent	
No	2.96 (1.55, 5.64)	0.001
Participated in peer education in the past year		
No	Referent	
Yes	1.56 (1.18, 2.07)	0.002

^a^Refers to adjusted odds ratio of length of sex work in the current location to HIV-related knowledge, HIV-related behaviour and access to preventive intervention services. HIV-related knowledge, HIV-related behaviour, and access to preventive intervention were input into the logistic regression model as dependent variables respectively. The length of sex work in current location was used as independent variable. Current age, type of working venues, marital status, nationality, years of education, the total duration of sex work, age when first engaged in commercial sex work, and average charge per sex transaction were used as covariates for adjustment, and adjusted OR were calculated

### Factors associated with HIV infection

As shown in Table 3, univariate analysis revealed that Vietnamese nationality (OR: 16.28, 95% CI 3.79–69.88, P < 0.001), average charge per sex transaction (OR: 0.96, 95% CI 0.95–0.98, P < 0.001), low-level of HIV-related knowledge (OR: 5.61, 95% CI 2.15–14.65, P < 0.001), inconsistent condom use in the past month (OR: 4.43, 95% CI 1.83–10.70, P = 0.001), more male clients in the past month (OR: 3.48, 95% CI 1.17–10.32, P = 0.025), no regular male clients in the past month (OR: 2.63, 95% CI 1.12–6.19, P = 0.027), and having a history of male clients who used aphrodisiacs (OR: 3.13, 95% CI 1.04–9.41, P = 0.042) were positively associated with HIV infection. Types of working venues, current age, marital status, years of education, total duration of sex work, length of sex work in current location, lifetime illicit drug use, receipt of free condom distribution and education/HIV counselling and testing programme in the past year, and participation in peer education in the past year did not show association with HIV status. After adjustment by all factors, Vietnamese nationality (OR: 6.70, 95% CI 1.16–38.77, P = 0.034) and no regular male clients in the past month (OR: 3.26, 95% CI 1.14–9.29, P = 0.027) exhibited increased risks for HIV infection, while ≥ 18 years when they first engaged in commercial sex work (OR: 0.03, 95% CI 0.01–0.57, P = 0.020) and higher average charge per sex transaction (OR: 0.97, 95% CI 0.95–0.99, P = 0.017) correlated with decreased risk for HIV infection.

**Table 3:** Unadjusted and adjusted analysis of factors associated with HIV infection

Independent variables	HIV infection (N = 22, %)	OR (95% *CI*)	P value	Adjusted OR (95% *CI*)	P value
Types of working venues					
Low-level	22 (100.0)	Referent		Referent	
Medium-level	0 (0.0)	NA	0.991	NA	0.990
High-level	0 (0.0)	NA	0.997	NA	0.997
Current age (years)	22 (100.0)	1.03 (0.98, 1.08)	0.201	1.01 (0.94, 1.09)	0.760
Marital status					
Unmarried/divorced/widowed	7 (31.8)	Referent		Referent	
Married/cohabiting	15 (68.2)	0.80 (0.33, 1.99)	0.637	0.60 (0.21, 1.71)	0.337
Nationality					
Chinese	2 (9.1)	Referent		Referent	
Vietnamese	20 (90.9)	16.28 (3.79, 69.88)	< 0.001	6.70 (1.16, 38.77)	0.034
Years of education					
≤ 9	21 (95.5)	Referent		Referent	
> 9	1 (4.5)	0.41 (0.06, 3.05)	0.382	1.72 (0.18, 16.70)	0.639
Total duration of sex work (months)	22 (100.0)	1.00 (0.99, 1.01)	0.394	1.01 (0.99, 1.02)	0.189
Age when they first engaged in commercial sex work (years)					
< 18	1 (4.5)	Referent		Referent	
≥ 18	21 (95.5)	0.54 (1.86, 4.08)	0.548	0.03 (0.01, 0.57)	0.020
Average charge per sex transaction (RMB)	22 (100.0)	0.96 (0.95, 0.98)	< 0.001	0.97 (0.95, 0.99)	0.017
Length of sex work in current location (months)					
≥ 6	14 (63.6)	Referent		Referent	
< 6	8 (36.4)	1.06 (0.44, 2.53)	0.905	0.67 (0.21, 2.11)	0.491
The level of HIV-related knowledge					
High-level	16 (92.7)	Referent		Referent	
Low-level	6 (27.3)	5.61 (2.15, 14.65)	< 0.001	1.27 (0.38, 4.28)	0.704
Consistent condom use in past month					
Yes	14 (63.6)	Referent		Referent	
No	8 (36.4)	4.43 (1.83, 10.70)	0.001	1.52 (0.50, 4.66)	0.465
The number of male clients in the past month					
≤ 30	4 (18.2)	Referent		Referent	
> 30	18 (81.8)	3.48 (1.17, 10.32)	0.025	1.21 (0.35, 4.18)	0.760
Had regular male clients in the past month					
Yes	9 (40.9)	Referent		Referent	
No	13 (59.1)	2.63 (1.12, 6.19)	0.027	3.26 (1.14, 9.29)	0.027
Had a history of male clients used aphrodisiacs					
No	18 (81.8)	Referent		Referent	
Yes	4 (18.2)	3.13 (1.04, 9.41)	0.042	2.51 (1.59, 3.94)	0.503
Lifetime illicit drug use					
Yes	0 (0.0)	Referent		Referent	
No	22 (100.0)	NA	1.000	NA	1.000
Received free condoms distribution and education/HIV counselling and testing program in the past year					
Yes	20 (90.9)	Referent		Referent	
No	2 (9.1)	2.84 (0.65, 12.44)	0.167	1.66 (0.27, 10.04)	0.581
Participated in peer education in the past year					
No	12 (54.5)	Referent		Referent	
Yes	10 (45.5)	0.47 (0.20, 1.09)	0.078	0.33 (0.11, 10.04)	0.058

*NA* not applicable

## Discussion

In our study, the rate of HIV-positive cases among all migrant FSWs was 1.32%, similar to those in studies conducted in other cities of Guangxi [20, 21]. Differences in demographics, HIV-related behaviours and access to preventive intervention services were found between the short-term work group and long-term work group. FSWs with short-term work in their current location were more likely to have risky sex but were less likely to receive health service resources. Moreover, FSWs with short-term work in their current location were associated HIV risk factors, including being a Vietnamese FSW, being under 18 years when they first engaged in commercial sex work and having no regular male clients. Working in a current location for a short time may be a potential risk for the spread of HIV/AIDS. Therefore, it is necessary to pay close attention to the risk behaviours and healthy status of FSWs who recently began working in a locality and to provide more access to health services for these FSWs.

Vietnamese FSWs had a higher risk of HIV infection, consistent with findings in other border regions in China [22]. Migrant Vietnamese FSWs are marginalized by society because of their illegal immigration status, and they don’t have the same access to heath services as local residents [10]. Moreover, our study found that Vietnamese FSWs were more likely to have short-term work in border regions. It is not clear why Vietnamese FSWs tended to do short-term work, which may be associated with unsafe working conditions caused by their illegal immigration status. Vietnamese female sex workers in border areas may face greater hazards of local police inquiries, fines, arrests and so on and have to change their workplaces frequently. Hence, Vietnamese FSWs, especially those who also had a short length of work in the current location, may be at increased risk of HIV transmission. As Vietnamese FSWs were more likely to do short-term work in their current location, regular monitoring of HIV status and mobility in cross-border female sex workers is required. Additionally, given their communication difficulties and lack of social connections, it is particularly important to strengthen peer education and provide more access to health service for these FSWs to reduce their high-risk behaviours.

Migrant FSWs with short-term work in their current location were more likely to be under 18 years old when they first engaged in commercial sex work, which was also a risk factor for HIV infection in our study. Beginning sex work earlier would affect their risk behaviour in adulthood [23, 24], and FSWs who initiated sex work at an early age endorsed less condom negotiation self-efficacy [23], leading to unprotected sex. There is a need for more professional health education and peer education for this group to improve their awareness of condom use and skills of condom negotiation.

A low charge for sex transactions was correlated with HIV infection. A poor financial condition is generally a powerful driving force for FSWs to engage in sex work. Most FSWs were in debt and had no financial support from others [25], and FSWs who have shorter average durations of stay were even more likely to be currently in debt [13]. Low charges for sex transaction may push FSWs to engage in more unprotected sex to meet the client’s needs for the purpose of alleviating economic pressures. In our study, FSWs working for a short time in their current location were at higher risk of inconsistent condom use than FSWs with long-term work, in accordance with the findings that FSWs with short durations of stay were more likely to use condoms inconsistently [13], and they had more male clients. Furthermore, due to their unfamiliarity in a new environment, FSWs with short-term work in their current location were less likely to have regular male clients. The lack of regular male clients increased the risk of HIV infection, consistent with previous research in Guangxi that male clients with no steady sexual partners had higher odds of HIV infection [26]. Overall, migrant FSWs with short-term work in their current location were at higher risk for HIV acquisition and may increase the risk of HIV/AIDS spreading. More research is needed to understand migrant FSWs’ working conditions, economic situations and access to and utilization of health services.

Furthermore, our study also found that ST FSWs had higher risks of having a history of male clients who used aphrodisiacs, which was seldom reported in previous studies. An aphrodisiacs is a medicine for erectile dysfunction. Due to their ability to induce intense sexual desire and prolong sexual intercourse, the use of aphrodisiacs is regarded as a marker of high-risk behaviour for HIV transmission [27]. In Guangxi, aphrodisiacs are widely used because of their low price [27]. Male clients in Guangxi were more likely to use aphrodisiacs when having sex with non-steady sexual partners [26], which puts them at a higher risk of HIV infection. Since aphrodisiacs are mostly used by elderly male clients in low-level commercial sex venues [27], not only female sex workers but also male clients need preventive interventions. In addition, more efforts should be made to publicize AIDS prevention and condom distribution in these venues.

Although many interventions and healthcare measures are provided for FSWs, it seldom consider the influence of migrant FSWs. In our study, compared with LT FSWs, ST FSWs were more likely to have a low level of HIV-related knowledge, and were less likely to receive free condoms distribution and education or HIV counselling and testing programme. The lack of access to preventive interventions and education for FSWs with short-term work in their current location resulted in low levels of HIV-related knowledge, which would affect their use of condoms [28]. Increasing access to preventive interventions for migrant FSWs is essential, and consideration should be given to targeted interventions for FSWs who recently began working locally. Moreover, in China’s current AIDS prevention policy, many free medical services are only available to Chinese citizens infected with HIV. In border areas, we may need local governments and medical institutions to focus on health resources for cross-border female sex workers.

We acknowledge several study limitations. First, our research only focused on the impact of work time after arrival to a current location on HIV prevalence and related behaviours, but the actual vulnerabilities faced by migrant FSWs are not known. More investigations on the correlation between vulnerabilities and work time are necessary. Second, our research was limited to two border cities in Guangxi. Data from a broader swath of the border region would help better understand the high-risk behaviour of migrant FSWs. In addition, our study is a cross-sectional study with no follow-up of ST FSWs, so the progress from their high-risk behaviour to infection with HIV could not be observed. Therefore, larger scale longitudinal studies such as cohort studies are needed to confirm the findings of this study.

## Conclusions

In conclusion, our study is the first examination of the relation between length of sex work in a current location and HIV-related behaviour among migrant FSWs. We found that short-term work in a current location correlated with HIV risk factors. A large number of migrant FSWs may make it hard to prevent the transmission of HIV. Considering that migrant FSWs with short-term work at their current location had fewer chances to acquire health resources, more targeted interventions should be proposed for FSWs who recently began working in a locality, especially cross-border migrant FSWs, to further prevent the spread of AIDS.

## Supplementary information

**Additional file 1: Table S1.** HIV-related behavior and access to preventive intervention services between the ST FSWs and LT FSWs. **Table S2.** Multivariate analysis of factors associated with low-level of HIV-related knowledge. **Table S3.** Multivariate analysis of factors associated with inconsistent condom use in the past month and lifetime illicit drug use. **Table S4.** Multivariate analysis of factors associated with male clients > 30 in the past month, no regular male clients in the past month and having a history of male clients used aphrodisiacs. **Table S5.** Multivariate analysis of factors associated with access to preventive intervention services.

## Data Availability

The datasets generated and/or analyzed during the current study are not publicly available due to ethical and legal reasons, but are available from the corresponding author on reasonable request.
