# Supplementary material for: Migrant female sex workers working at the Sino-Vietnamese border for a short time have a higher risk of HIV transmission: a consecutive cross-sectional study
Source: AIDS Res Ther. 2020 Feb 7;17:4. doi: 10.1186/s12981-020-0260-0 (PMC7006200; doi:10.1186/s12981-020-0260-0)
Supplement: Supplementary file 1 — Additional file 1: Table S1. HIV-related behavior and access to preventive intervention services between the ST FSWs and LT FSWs. Table S2. Multivariate analysis of factors associated with low-level of HIV-related knowledge. Table S3. Multivariate analysis of factors associated with inconsistent condom use in the past month and lifetime illicit drug use. Table S4. Multivariate analysis of factors associated with male clients > 30 in the past month, no regular male clients in the past month and having a history of male clients used aphrodisiacs. Table S5. Multivariate analysis of factors associated with access to preventive intervention services. [file 12981_2020_260_MOESM1_ESM.doc]

**Table S1** HIV-related behavior and access to preventive intervention services between the ST FSWs and LT FSWs

| Variables | Total  (N=1667, %) | ST FSWs (N=586, %) | LT FSWs (N=1081, %) | *Χ^2^/t* | P |
| --- | --- | --- | --- | --- | --- |
| Consistent condom use in the past month |  |  |  | 26.135 | <0.001 |
| Yes | 1471 (88.2) | 485 (82.8) | 986 (91.2) |  |  |
| No | 196 (11.8) | 101 (17.2) | 95 (8.8) |  |  |
| The number of male clients in the past month |  |  |  | -8.826* | <0.001 |
| Median (IQR) | 1667 (100%) | 30 (48, 64) | 25 (15, 55) |  |  |
| Had regular male clents in the past month |  |  |  | 41.917 | <0.001 |
| Yes | 1071 (64.2) | 316 (53.9) | 755 (69.8) |  |  |
| No | 596 (35.8) | 270 (46.1) | 326 (30.2) |  |  |
| Had a history of male clients used aphrodisiacs during sex |  |  |  | 12.431 | <0.001 |
| No | 1554 (93.2) | 529 (90.3) | 1025 (94.8) |  |  |
| Yes | 113 (6.8) | 57 (9.7) | 56 (5.2) |  |  |
| Lifetime illicit drug use |  |  |  | 0.063 | 0.802 |
| Yes | 13 (0.8) | 5 (0.9) | 8 (0.7) |  |  |
| No | 1654 (99.2) | 581 (99.1) | 1073 (99.3) |  |  |
| Received free condom distribution and education / HIV counseling & testing program in the past year |  |  |  | 16.729 | <0.001 |
| Yes | 58 (3.5) | 35 (6.0) | 23 (2.1) |  |  |
| No | 1609 (96.5) | 551 (94.0) | 1058 (97.9) |  |  |
| Participated in peer education in the past year |  |  |  | 2.908 | 0.088 |
| Yes | 1064 (63.8) | 390 (66.6) | 674 (62.4) |  |  |
| No | 603 (36.2) | 196 (33.4) | 407 (37.6) |  |  |

* Refers to corrected t test

Table S2 Multivariate analysis of factors associated with low-level of HIV-related knowledge.

| Independent variables | Low-level of HIV-related knowledge (N=109, %) | Adjusted OR (95% *CI*) | P value |
| --- | --- | --- | --- |
| Types of working venues |  |  |  |
| Low-level | 90 (82.6) | Referent |  |
| Medium-level | 16 (14.6) | 0.24 (0.13, 0.45) | <0.001 |
| High-level | 3 (2.8) | 0.64 (0.18, 2.34) | 0.498 |
| Current age (years) | 109 (100.0) | 1.03 (0.99, 1.06) | 0.106 |
| Marital status |  |  |  |
| [Unmarried](#/javascript:;) / [divorced](#/javascript:;) / widowed | 40 (36.7) | Referent |  |
| Married / Cohabiting | 69 (63.3) | 0.68 (0.42, 1.10) | 0.114 |
| Nationality |  |  |  |
| Chinese | 33 (30.3) | Referent |  |
| Vietnamese | 76 (69.7) | 3.80 (2.17, 6.65) | <0.001 |
| Years of [education](#/javascript:;) |  |  |  |
| ≤ 9 | 109 (100.0) | Referent |  |
| > 9 | 0 (0.0) | NA | 0.995 |
| Total duration of sex work (months) | 109 (100.0) | 1.00 (0.99, 1.01) | 0.452 |
| Age when they first engaged in commercial sex work (years) |  |  |  |
| < 18 | 2 (1.8) | Referent |  |
| ≥ 18 | 107 (98.2) | 1.74 (0.38, 8.03) | 0.476 |
| Average charge per sex transaction (RMB) | 109 (100.0) | 1.01 (0.99, 1.02) | 0.081 |
| Length of sex work in current location (months) |  |  |  |
| ≥ 6 | 49 (45.0) | Referent |  |
| < 6 | 60 (55.0) | 3.35 (2.11, 5.32) | <0.001 |

**Table S3** Multivariate analysis of factors associated with inconsistent condom use in the past month and lifetime illicit drug use

| Independent variables | Inconsistent condom use in the past month | | |  | Lifetime illicit drug use | | |
| --- | --- | --- | --- | --- | --- | --- | --- |
|  | N=196 (%) | Adjusted OR (95% *CI*) | P value |  | N=13 (%) | Adjusted OR (95% *CI*) | P value |
| Types of working venues |  |  |  |  |  |  |  |
| Low-level | 152 (77.6) | Referent |  |  | 5 (38.5) | Referent |  |
| Medium-level | 40 (20.4) | 0.58 (0.37, 0.91) | 0.018 |  | 8 (61.5) | 0.76 (0.20, 2.92) | 0.693 |
| High-level | 4 (2.0) | 1.73 (0.54, 5.52) | 0.359 |  | 0 (0.0) | NA | 0.997 |
| Current age (years) | 196 (100.0) | 1.05 (1.03, 1.08) | <0.001 |  | 13 (100.0) | 1.09 (0.98, 1.21) | 0.126 |
| Marital status |  |  |  |  |  |  |  |
| [Unmarried](#/javascript:;) / [divorced](#/javascript:;) / widowed | 55 (28.1) | Referent |  |  | 8 (61.5) | Referent |  |
| Married / Cohabiting | 141 (71.9) | 0.58 (0.39, 0.85) | 0.006 |  | 5 (38.5) | 2.23 (0.60, 8.28) | 0.230 |
| Nationality |  |  |  |  |  |  |  |
| Chinese | 92 (46.9) | Referent |  |  | 8 (61.5) | Referent |  |
| Vietnamese | 104 (53.1) | 1.22 (0.79, 1.88) | 0.364 |  | 5 (38.5) | 1.05 (0.24, 4.61) | 0.951 |
| Years of [education](#/javascript:;) |  |  |  |  |  |  |  |
| ≤ 9 | 180 (91.8) | Referent |  |  | 11 (84.6) | Referent |  |
| > 9 | 16 (8.2) | 0.62 (0.34, 1.13) | 0.118 |  | 2 (15.4) | 0.61 (0.12, 3.08) | 0.549 |
| Total duration of sex work (months) | 196 (100.0) | 1.00 (0.99, 1.01) | 0.442 |  | 13 (100.0) | 0.99 (0.98, 1.01) | 0.107 |
| Age when they first engaged in commercial sex work (years) |  |  |  |  |  |  |  |
| < 18 | 7 (3.6) | Referent |  |  | 12 (92.3) | Referent |  |
| ≥ 18 | 189 (96.4) | 0.29 (0.11, 0.77) | 0.013 |  | 1 (7.7) | 0.62 (0.06, 6.06) | 0.677 |
| Average charge per sex transaction (RMB) | 196 (100.0) | 0.98 (0.97, 0.98) | <0.001 |  | 13 (100.0) | 0.99 (0.98, 1.01) | 0.206 |
| Length of sex work in current location |  |  |  |  |  |  |  |
| ≥ 6 months | 95 (48.5) | Referent |  |  | 8 (61.5) | Referent |  |
| < 6 months | 101 (51.5) | 2.94 (2.03, 4.24) | <0.001 |  | 5 (38.5) | 1.21 (0.32, 4.54) | 0.778 |

NA Refers to not available.

**Table S4** Multivariate analysis of factors associated with male clients >30 in the past month, no regular male clients in the past month and having a history of male clients used aphrodisiacs

| Independent variables | Male clients >30 in the past month | | |  | No regular male clients in the past month | | |  | Had a history of male clients used aphrodisiacs | | |
| --- | --- | --- | --- | --- | --- | --- | --- | --- | --- | --- | --- |
|  | N=721 (%) | Adjusted OR (95% *CI*) | P value |  | N=596 (%) | Adjusted OR (95% *CI*) | P value |  | N=113 (%) | Adjusted OR (95% *CI*) | P value |
| Types of working venues |  |  |  |  |  |  |  |  |  |  |  |
| Low-level | 497 (68.9) | Referent |  |  | 320 (53.7) | Referent |  |  | 44 (38.9) | Referent | <0.001 |
| Medium-level | 207 (28.7) | 2.49 (1.82, 3.40) | <0.001 |  | 234 (39.3) | 0.88 (0.66, 1.11) | 0.241 |  | 67 (59.3) | 3.62 (2.20, 5.96) | <0.001 |
| High-level | 17 (2.4) | 4.88 (2.52, 9.46) | <0.001 |  | 42 (7.0) | 2.18 (1.23, 3.89) | 0.008 |  | 2 (1.8) | 1.83 (0.39, 8.61) | 0.447 |
| Current age (years) | 721 (100.0) | 0.92 (0.90, 0.93) | <0.001 |  | 596 (100.0) | 0.98 (0.97, 1.00) | 0.035 |  | 113 (100.0) | 1.05 (1.02, 1.08) | 0.002 |
| Marital status |  |  |  |  |  |  |  |  |  |  |  |
| [Unmarried](#/javascript:;) / [divorced](#/javascript:;) / widowed | 121 (16.8) | Referent |  |  | 192 (32.2) | Referent |  |  | 29 (25.7) | Referent |  |
| Married / Cohabiting | 600 (83.2) | 0.64 (0.46, 0.87) | 0.005 |  | 404 (67.8) | 0.92 (0.71, 1.19) | 0.524 |  | 84 (74.3) | 0.72 (0.44, 1.18) | 0.198 |
| Nationality |  |  |  |  |  |  |  |  |  |  |  |
| Chinese | 627 (87.0) | Referent |  |  | 354 (59.4) | Referent |  |  | 74 (65.5) | Referent |  |
| Vietnamese | 94 (13.0) | 13.53 (9.43, 19.43) | <0.001 |  | 242 (40.6) | 0.91 (0.69, 1.21) | 0.522 |  | 39 (34.5) | 1.23 (0.72, 2.09) | 0.449 |
| Years of [education](#/javascript:;) |  |  |  |  |  |  |  |  |  |  |  |
| ≤ 9 | 673 (93.3) | Referent |  |  | 540 (90.6) | Referent |  |  | 102 (90.3) | Referent |  |
| > 9 | 48 (6.7) | 0.94 (0.59, 1.49) | 0.778 |  | 56 (9.4) | 0.56 (0.39, 0.80) | 0.002 |  | 11 (9.7) | 0.67 (0.34, 1.35) | 0.264 |
| Total duration of sex work (months) | 721 (100.0) | 1.01 (1.00, 1.020) | <0.001 |  | 596 (100.0) | 1.00 (0.99, 1.01) | 0.125 |  | 113 (100.0) | 1.01 (1.00, 1.02) | <0.001 |
| Age when they first engaged in commercial sex work (years) |  |  |  |  |  |  |  |  |  |  |  |
| < 18 | 3 (0.4) | Referent |  |  | 19 (3.2) | Referent |  |  | 1 (0.9) | Referent |  |
| ≥ 18 | 718 (99.6) | 0.77 (0.21, 2.78) | 0.687 |  | 577 (96.8) | 1.39 (0.71, 2.72) | 0.331 |  | 112 (99.1) | 2.79 (0.36, 21.80) | 0.329 |
| Average charge per sex transaction (RMB) | 721 (100.0) | 1.00 (0.99, 1.01) | 0.170 |  | 596 (100.0) | 1.01 (1.00, 1.02) | 0.051 |  | 113 (100.0) | 0.99 (0.98, 1.00) | 0.140 |
| Length of sex work in current location |  |  |  |  |  |  |  |  |  |  |  |
| ≥ 6 months | 570 (79.1) | Referent |  |  | 326 (54.7) | Referent |  |  | 56 (49.6) | Referent |  |
| < 6 months | 151 (20.9) | 1.68 (1.24, 2.26) | 0.001 |  | 270 (45.3) | 2.03 (1.58, 2.60) | <0.001 |  | 57 (50.4) | 2.51 (1.59, 3.94) | <0.001 |

NA Refers to not available.

**Table S5** Multivariate analysis of factors associated with access to preventive intervention services

| Independent variables | No receiving free condom distribution and education / HIV counseling & testing program in the past year | | |  | Participation in peer education in the past year | | |
| --- | --- | --- | --- | --- | --- | --- | --- |
|  | N=58 (%) | Adjusted OR (95% *CI*) | P value |  | N=603 (%) | Adjusted OR (95% *CI*) | P value |
| Types of working venues |  |  |  |  |  |  |  |
| Low-level | 35 (60.3) | Referent |  |  | 269 (44.6) | Referent |  |
| Medium-level | 21 (36.2) | 0.50 (0.25, 0.99) | 0.045 |  | 318 (52.7) | 0.39 (0.30, 0.51) | <0.001 |
| High-level | 2 (3.5) | 0.33 (0.07, 1.64) | 0.176 |  | 16 (2.7) | 2.63 (1.37, 5.05) | 0.004 |
| Current age (years) | 58 (100.0) | 1.030 (0.99, 1.08) | 0.184 |  | 603 (100.0) | 1.03 (1.01, 1.05) | 0.003 |
| Marital status |  |  |  |  |  |  |  |
| [Unmarried](#/javascript:;) / [divorced](#/javascript:;) / widowed | 29 (50.0) | Referent |  |  | 202 (33.5) | Referent |  |
| Married / Cohabiting | 29 (50.0) | 0.63 (0.33, 1.21) | 0.167 |  | 401 (66.5) | 1.16 (0.87, 1.58) | 0.310 |
| Nationality |  |  |  |  |  |  |  |
| Chinese | 23 (39.7) | Referent |  |  | 380 (63.0) | Referent |  |
| Vietnamese | 35 (60.3) | 3.63 (1.72, 7.63) | 0.001 |  | 223 (37.0) | 0.57 (0.42, 0.78) | <0.001 |
| Years of [education](#/javascript:;) |  |  |  |  |  |  |  |
| ≤ 9 | 57 (98.3) | Referent |  |  | 577 (95.7) | Referent |  |
| > 9 | 1 (1.7) | 0.08 (0.01, 0.63) | 0.017 |  | 26 (4.3) | 5.72 (3.56, 9.20) | <0.001 |
| Total duration of sex work (months) | 58 (100.0) | 0.98 (0.97, 0.99) | 0.036 |  | 603 (100.0) | 0.99 (0.98, 1.01) | 0.225 |
| Age when first engaged in commercial sex work (years) |  |  |  |  |  |  |  |
| < 18 | 1 (1.7) | Referent |  |  | 588 (97.5) | Referent |  |
| ≥ 18 | 57 (98.3) | 7.85 (0.93, 66.07) | 0.058 |  | 15 (2.5) | 0.22 (0.10, 0.46) | <0.001 |
| Average charge per sex transaction (RMB) | 58 (100.0) | 1.03 (1.02, 1.04) | 0.000 |  | 603 (100.0) | 0.98 (0.98, 0.99) | <0.001 |
| Length of sex work in current location |  |  |  |  |  |  |  |
| ≥ 6 months | 23 (39.7) | Referent |  |  | 407 (67.5) | Referent |  |
| < 6 months | 35 (60.3) | 2.96 (1.55, 5.64) | 0.001 |  | 196 (32.5) | 1.56 (1.18, 2.07) | 0.002 |
